# Mendelian randomization study of maternal coffee consumption and its influence on birthweight, stillbirth, miscarriage, gestational age and pre-term birth

**DOI:** 10.1093/ije/dyac121

**Published:** 2022-06-09

**Authors:** Caroline Brito Nunes, Peiyuan Huang, Geng Wang, Mischa Lundberg, Shannon D’Urso, Robyn E Wootton, Maria Carolina Borges, Deborah A Lawlor, Nicole M Warrington, David M Evans, Liang-Dar Hwang, Gunn-Helen Moen

**Affiliations:** School of Biomedical Sciences, Faculty of Medicine, The University of Queensland, Brisbane, Australia; Medical Research Council Integrative Epidemiology Unit at the University of Bristol, Bristol, UK; Population Health Science, Bristol Medical School, University of Bristol, Bristol, UK; The University of Queensland Diamantina Institute, The University of Queensland, Brisbane, Australia; The University of Queensland Diamantina Institute, The University of Queensland, Brisbane, Australia; Institute for Molecular Bioscience, The University of Queensland, Brisbane, Australia; Medical Research Council Integrative Epidemiology Unit at the University of Bristol, Bristol, UK; Population Health Science, Bristol Medical School, University of Bristol, Bristol, UK; Nic Waals Institute, Lovisenberg Diaconal Hospital, Oslo, Norway; Medical Research Council Integrative Epidemiology Unit at the University of Bristol, Bristol, UK; Population Health Science, Bristol Medical School, University of Bristol, Bristol, UK; Medical Research Council Integrative Epidemiology Unit at the University of Bristol, Bristol, UK; Population Health Science, Bristol Medical School, University of Bristol, Bristol, UK; The University of Queensland Diamantina Institute, The University of Queensland, Brisbane, Australia; Institute for Molecular Bioscience, The University of Queensland, Brisbane, Australia; Department of Public Health and Nursing, K.G. Jebsen Center for Genetic Epidemiology, NTNU, Norwegian University of Science and Technology, Trondheim, Norway; Medical Research Council Integrative Epidemiology Unit at the University of Bristol, Bristol, UK; The University of Queensland Diamantina Institute, The University of Queensland, Brisbane, Australia; Institute for Molecular Bioscience, The University of Queensland, Brisbane, Australia; The University of Queensland Diamantina Institute, The University of Queensland, Brisbane, Australia; Institute for Molecular Bioscience, The University of Queensland, Brisbane, Australia; Population Health Science, Bristol Medical School, University of Bristol, Bristol, UK; The University of Queensland Diamantina Institute, The University of Queensland, Brisbane, Australia; Institute for Molecular Bioscience, The University of Queensland, Brisbane, Australia; Department of Public Health and Nursing, K.G. Jebsen Center for Genetic Epidemiology, NTNU, Norwegian University of Science and Technology, Trondheim, Norway; Institute of Clinical Medicine, Faculty of Medicine, University of Oslo, Oslo, Norway

**Keywords:** Mendelian randomization, coffee, maternal genetic effect, birthweight, stillbirth, miscarriage, gestational age, pre-term birth, ALSPAC, UK Biobank

## Abstract

**Background:**

Coffee consumption has been associated with several adverse pregnancy outcomes, although data from randomized–controlled trials are lacking. We investigate whether there is a causal relationship between coffee consumption and miscarriage, stillbirth, birthweight, gestational age and pre-term birth using Mendelian randomization (MR).

**Methods:**

A two-sample MR study was performed using summary results data from a genome-wide association meta-analysis of coffee consumption (*N* = 91 462) from the Coffee and Caffeine Genetics Consortium. Outcomes included self-reported miscarriage (*N* = 49 996 cases and 174 109 controls from a large meta-analysis); the number of stillbirths [*N* = 60 453 from UK Biobank (UKBB)]; gestational age and pre-term birth (*N* = 43 568 from the 23andMe, Inc cohort) and birthweight (*N* = 297 356 reporting own birthweight and *N* = 210 248 reporting offspring’s birthweight from UKBB and the Early Growth Genetics Consortium). Additionally, a one-sample genetic risk score (GRS) analysis of coffee consumption in UKBB women (*N* up to 194 196) and the Avon Longitudinal Study of Parents and Children (*N* up to 6845 mothers and 4510 children) and its relationship with offspring outcomes was performed.

**Results:**

Both the two-sample MR and one-sample GRS analyses showed no change in risk of sporadic miscarriages, stillbirths, pre-term birth or effect on gestational age connected to coffee consumption. Although both analyses showed an association between increased coffee consumption and higher birthweight, the magnitude of the effect was inconsistent.

**Conclusion:**

Our results suggest that coffee consumption during pregnancy might not itself contribute to adverse outcomes such as stillbirth, sporadic miscarriages and pre-term birth or lower gestational age or birthweight of the offspring.

Key Messages

- Both weighted and unweighted maternal genetic risk scores for higher coffee consumption were associated with elevated coffee consumption at Week 32 of gestation.
- Maternal coffee consumption during pregnancy may not contribute to adverse outcomes such as stillbirth, sporadic miscarriages and pre-term birth.
- Maternal coffee consumption during pregnancy may influence birthweight of the offspring.

## Introduction

Coffee is the most consumed beverage worldwide, with an average daily intake of >400 mg of caffeine, equivalent to approximately four cups,^1^ per capita in many European countries, such as Norway, Sweden and Netherlands.^2^ Coffee is the primary source of caffeine in most populations, but other beverages such as tea and soft drinks, or foods such as chocolate, can also contribute to an individual’s total caffeine intake.^2^^,^^3^

It has been estimated that ∼70% of pregnant women in the USA consume caffeine during pregnancy, with coffee being the main source of caffeine.^3^ Several physiological changes occur during pregnancy that decrease the rate at which caffeine is metabolized—especially in the third trimester—due to decreased activity of the liver enzyme CYP1A.^4^ Therefore, caffeine can accumulate in the body throughout the pregnancy, with its half-life increasing from an average of 3 h for non-pregnant women to ≤18 h for pregnant women at the end of pregnancy.^5^^,^^6^ Since caffeine can freely cross the placenta and the fetus is unable to metabolize the molecule, the fetus is exposed to caffeine and its metabolites in proportion to levels consumed by the mother.^7^

The current World Health Organization guidelines recommend a caffeine intake of <300 mg/day during pregnancy,^8^ whereas the American College of Obstetricians and Gynecologists recommends a maximum caffeine intake of 200 mg/day,^9^ with one cup of coffee typically containing between 70 and 140 mg of caffeine depending on the type of bean and coffee, the degree of roasting and the serving size.^10^ A Scandinavian expert committee concluded that caffeine exposure during pregnancy should be limited due to a potential increased risk of spontaneous miscarriage and fetal growth restriction.^11^ However, they emphasized the lack of clear evidence of an association between coffee consumption and adverse outcomes, such as low birthweight,^12–15^ stillbirths^14^ and miscarriages,^14–16^ as many of the prior observational studies failed to address the potential confounding effects of smoking and alcohol intake during pregnancy.^14^^,^^17^

**Figure 1:**
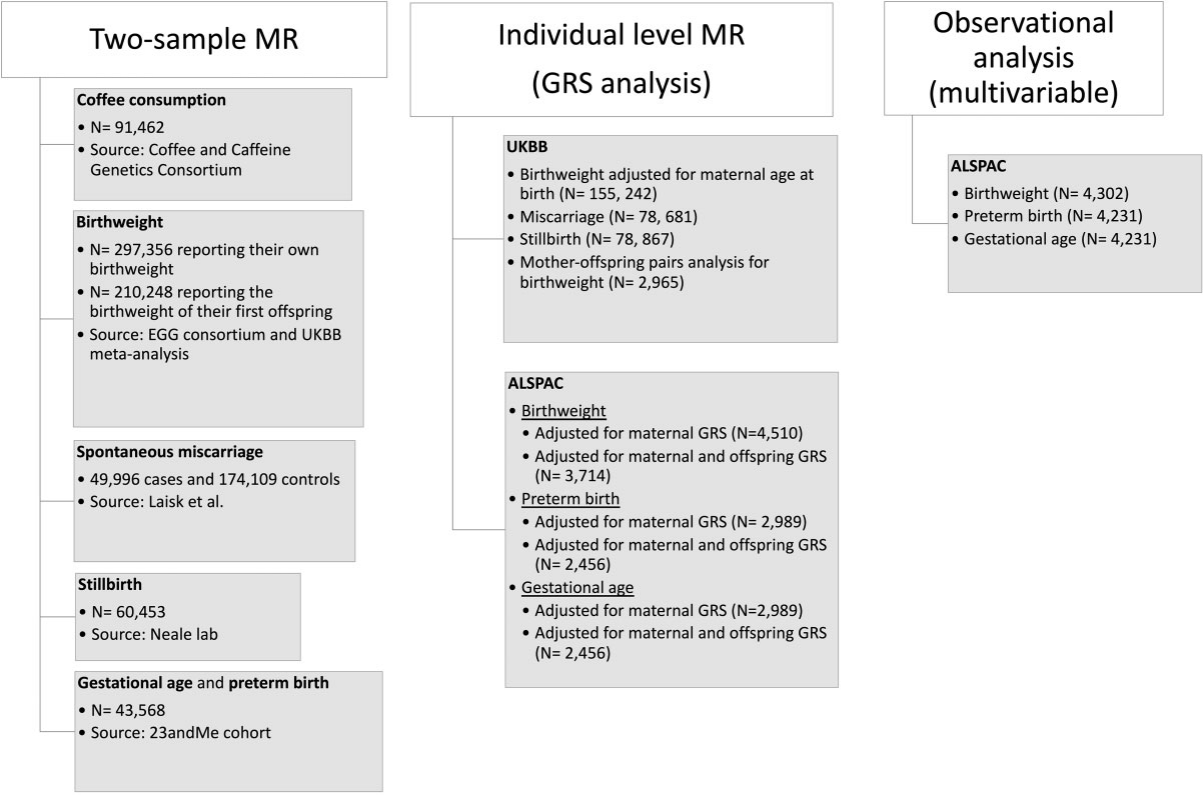
Overview of data sources and phenotypes used in the two-sample MR, individual-level MR and traditional observational analysis. GRS, genetic risk score; MR, Mendelian randomization; UKBB, UK Biobank; ALSPAC, Avon Longitudinal Study of Parents and Children; EEG, Early Growth Genetics.

Given it would be infeasible and/or unethical to randomize women to consume different levels of coffee during pregnancy, alternative methods for addressing the possible causal effect of maternal coffee consumption on pregnancy outcomes are needed. Mendelian randomization (MR), which uses genetic variants related to an exposure of interest as instruments for that exposure, is less prone to confounding by social, environmental and behavioural factors that affect many traditional observational epidemiological studies.^18–20^ In this paper, we use MR to investigate whether the observational relationships between coffee consumption and adverse pregnancy outcomes are causal. Specifically, we investigate the causal relationship between maternal coffee consumption and miscarriages, stillbirths, pre-term birth, gestational age and birthweight using two-sample summary data MR analysis.^21^ Additionally, we perform a one-sample genetic risk score (GRS) analysis using individual participant data from two large UK studies: UK Biobank (UKBB)^22^ and the Avon Longitudinal Study of Parents and Children (ALSPAC)^23^^,^^24^ (Figure 1).

## Methods

Genetic instruments were identified in a genome-wide association study (GWAS) of coffee consumption^25^ from the Coffee and Caffeine Genetics Consortium. We used a two-sample MR approach to assess the effect of these genetic instruments of coffee consumption on pregnancy outcomes using GWAS of birthweight,^26^ miscarriages,^27^ stillbirths (http://www.nealelab.is/uk-biobank; 10 December 2020, date last accessed) and gestational age and pre-term birth from 23andMe, Inc.^28^ Additionally, we performed an individual-level MR analysis using GRS for offspring birthweight, gestational age and pre-term birth in ALSPAC and for offspring birthweight, number of miscarriages and number of stillbirths in UKBB using linear regression. Finally, we assessed the association between coffee consumption in pregnancy and offspring birthweight, gestational age and risk of pre-term birth in ALSPAC using multivariable regression (for an overview, see Figure 1 and Supplementary Table S1, available as Supplementary data at *IJE* online). Detailed descriptions of these analyses are listed below in the sections ‘Two-sample MR analyses’, ‘Individual-level data MR analysis using GRS (ALSPAC and UKBB)’ and ‘Associations between self-reported coffee consumption and pregnancy outcomes in the ALSPAC study’.

### Two-sample MR analyses

#### Data sources

##### Coffee consumption

Eight single-nucleotide polymorphisms (SNPs) that were significantly associated with coffee consumption were extracted from summary results data from a GWAS of coffee consumption published by the Coffee and Caffeine Genetics Consortium (*N* = 91 462 individuals of European ancestry).^25^ The study reports a GWAS meta-analysis of coffee consumption (cups/day) across 28 population-based studies. The SNPs included in the MR analyses in this paper are listed in Supplementary Table S2 (available as Supplementary data at *IJE* online).

##### Birthweight

Summary results statistics from the GWAS of birthweight were obtained from the Early Growth Genetics (EGG) consortium and UKBB meta-analysis (maternal-specific genetic effects on offspring birthweight) (https://egg-consortium.org/birth-weight-2019.html).^26^ The birthweight GWAS included 297 356 individuals reporting their own birthweight and 210 248 females reporting the birthweight of their first offspring. A structural equation model was used to partition the genetic effects into maternal-specific and fetal-specific genetic effects on birthweight. Only the maternal-specific genetic effects on birthweight were used in the present study because the aim was to assess whether offspring birthweight was causally affected by maternal coffee consumption.

##### Miscarriage

Summary results statistics for sporadic miscarriages were obtained from Laisk *et al.*,^27^ who performed GWAS on 49 996 females of European ancestry who self-reported miscarriages and 174 109 female controls. Sporadic miscarriage was defined as one or two (self-reported) miscarriages.

##### Stillbirth

Summary results statistics from the GWAS of the number of self-reported stillbirths were obtained from publicly available summary statistics published by the Neale lab (http://www.nealelab.is/uk-biobank; 10 December 2020, date last accessed) that involved 60 453 women from UKBB who reported the number of stillbirths they experienced.

##### Gestational age and pre-term birth

GWAS summary results statistics for gestational age and pre-term birth (defined as a birth at <37 weeks of gestation) were obtained from 23andMe. These results contributed to the discovery data set for a previously published GWAS by Zhang *et al.*^28^ The original GWAS analysis included 43 568 unrelated women of European ancestry from 23andMe who self-reported length of gestation (in weeks) from their first live singleton birth. The full GWAS summary statistics for the 23andMe discovery data set were made available through 23andMe, under an agreement that protects the privacy of the 23andMe participants. Please visit https://research.23andme.com/collaborate/#dataset-access/ for more information and to apply to access the data.

#### Genetic instruments

We selected three sets of SNPs to instrument coffee intake (Supplementary Table S2, available as Supplementary data at *IJE* online). First, we selected eight independent SNPs that reached genome-wide significance in the GWAS of coffee consumption mentioned above (rs1481012, rs4410790, rs9902453, rs7800944, rs17685, rs2472297, rs1260326 and rs6265).^25^ Second, we kept six out of the eight SNPs for which there was no strong evidence of horizontal pleiotropy based on the results of a phenome-wide scan using PhenoScanner^29^^,^^30^ at a *P*-value threshold of 5 × 10^−^^8^. The *GCKR* variant (rs1260326) was associated with alcohol intake frequency, type 2 diabetes, fasting glucose levels, triglycerides levels, cardiovascular disease risk factors and chronic kidney disease, and the SNP rs6265 in the *BDNF* gene had previously been associated with several traits, including body mass index, weight, body fat and smoking (Supplementary Table S2, available as Supplementary data at *IJE* online).

Further, we tested the association between each of the eight SNPs and alcohol consumption and smoking behaviour in 194 196 UKBB women who reported either offspring birthweight (*N* = 173 259), having a stillbirth (*N* = 78 867) or having a spontaneous miscarriage (*N* = 78 681). Five out of eight SNPs were associated with either alcohol consumption or smoking behaviour, after Bonferroni correction (*P *<* *0.05/8 = 0.00625) (Supplementary Table S3, available as Supplementary data at *IJE* online). Therefore, for the third subset of SNPs, we kept three out of the eight SNPs (rs1481012, rs4410790 and rs9902453), which were not associated with smoking or alcohol intake in UKBB and had no strong evidence for pleiotropy in the above search in PhenoScanner^29^^,^^30^ (Supplementary Table S2, available as Supplementary data at *IJE* online). This analysis was performed because the GRS for coffee consumption had been found to be associated with smoking behaviour and alcohol intake frequency in UKBB previously^31^—two phenotypes strongly associated with adverse pregnancy outcomes.

#### Statistical analysis

A two-sample inverse-variance weighted (IVW) MR analysis was conducted using either eight, six or three genetic variants as explained above to estimate the causal relationship of maternal coffee consumption on sporadic miscarriages, stillbirths, gestational age, pre-term birth and offspring birthweight. All SNPs and their effect sizes for these phenotypes are listed in Supplementary Table S4 (available as Supplementary data at *IJE* online). A heterogeneity test of causal effect estimates was conducted across each of the variants for coffee consumption using Cochran’s Q. Directional pleiotropy was tested by assessing whether the MR–Egger intercept was different from zero. Sensitivity analyses were performed using MR–Egger regression, weighted median, weighted and simple mode estimation approaches. *I*^2^_GX_ was calculated to assess the potential for weak instrument bias in the MR–Egger regression analyses. However, due to the small number of variants used in the MR analyses, the ability of these methods to control for latent horizontal pleiotropy is likely to be limited. Bonferroni correction of *P*-value for the two-sample MR analysis was 0.05/5 phenotypes tested = 0.01.

A flowchart showing the number of individuals included in each analysis is presented in Supplementary Figure S1 (available as Supplementary data at *IJE* online). The TwoSampleMR package^32^ in R version 3.4.3 was used to perform all the analyses.

### Individual-level data MR analysis using GRS (ALSPAC and UKBB)

#### Data sources

##### UKBB

UKBB is a large prospective population-based cohort containing ∼500 000 individuals (aged 37–73 years; 54% female), representing 5.5% of those who were invited to participate.^22^ UKBB has ethical approval from the North West Multi-Centre Research Ethics Committee, which covers the UK, and all participants provided written informed consent. Up to 251 058 European women were available for analysis; a detailed description of the selection of these women is provided in Supplementary Note S1 (available as Supplementary data at *IJE* online).

We removed UKBB women of European ancestry who did not report any information on their offspring's birthweight (*N* = 20 937), stillbirth (*N* = 115 329) or spontaneous miscarriage (*N* = 115 329), leaving a total of 194 196 women who reported any of the birth outcomes along with their coffee consumption habit. The exact number of women who reported the number of stillbirths and miscarriages was 78 867 and 78 681, respectively. Out of the 194 196 women, only a total of 155 242 reported both their offspring birthweight and maternal age at birth. A detailed description of the phenotypes is presented in Supplementary Note S2 (available as Supplementary data at *IJE* online).

##### ALSPAC

ALSPAC is a longitudinal birth cohort study established to understand how genetic and environmental factors influence health and development in parents and children.^23^^,^^24^ A detailed description of the cohort is presented in Supplementary Note S3 (available as Supplementary data at *IJE* online).

Birthweight data for each child in the study were ascertained in a variety of ways, including from obstetric data records, recorded by the ALSPAC research team and from the birth notification service in the UK. If the birthweight measurement for an individual differed across the different sources (defined as >100 grams difference), the individual was excluded from the analysis. When the differences were ≤100 grams, the lower birthweight measurement was used. In the study, we excluded offspring with birthweight of <2.5 or >4.5 kg to match the inclusion criterion of the GWAS of birthweight. Gestational age was defined as the weeks of gestation based on the final clinically estimated date of delivery and pre-term birth was defined as <37 weeks of gestation.

Maternal coffee intake was defined as cups of coffee per day at Week 32 of pregnancy (*N* = 6845). Maternal alcohol drinking was derived from units of alcohol drunk per week at Week 32 of pregnancy, with those who did not drink defined as non-drinkers (*N* = 2826) and the rest defined as drinkers (*N* = 1525). Maternal smoking was derived from the smoking behaviour in the first trimester of pregnancy, with those smoking any types of tobacco defined as smokers (*N* = 1620) and the rest defined as non-smokers (*N* = 5702). These data were all collected from a self-reported questionnaire.

#### Genetic instruments

We constructed both weighted and unweighted GRSs using the three sets of SNPs mentioned above in the two-sample MR section, resulting in six different GRSs for coffee consumption. We constructed an unweighted GRS as the regression coefficients obtained in the coffee consumption GWAS might not accurately reflect effect sizes during pregnancy. These GRSs explained approximately 1.94% (eight SNPs), 1.81% (six SNPs) and 0.82% (three SNPs) of the variance in coffee drinking according to the effect estimates reported in the exposure GWAS.^25^ The weighted risk scores were created by multiplying the coffee-increasing alleles by their effect size from the exposure GWAS whereas the unweighted risk scores were obtained by adding the number of coffee consumption increasing alleles together.^25^

#### Statistical analysis

##### UKBB

Linear regression analyses were used to assess the relationship between the maternal GRSs for coffee consumption (both weighted and unweighted) and self-reported and maternal coffee intake (cups/day) in addition to the number of spontaneous miscarriages, number of stillbirths and birthweight of the first offspring, adjusting for maternal age at birth (to reflect age at the pregnancy in question). Maternal age was only available for birthweight as women only reported their total number of miscarriages or stillbirths. To match the inclusion criterion of the GWAS of birthweight, if the birthweight of the offspring was <5 or >10 pounds (equivalent to approximately <2.3 or >4.5 kg), the value was excluded from the analysis. Gestational age and pre-term birth were only available in a small subsample in UKBB, and therefore were not included in the analyses here.

Additionally, we utilized the mother–offspring pairs in UKBB and performed a linear regression of maternal unweighted GRS for coffee consumption and offspring own-reported birthweight, adjusting for offspring unweighted GRS. We also conducted linear regression of maternal weighted GRS for coffee consumption on offspring own-reported birthweight, adjusting for individual offspring SNPs.^33^ A description of the mother–offspring pairs is provided in Supplementary Note S4 (available as Supplementary data at *IJE* online).

One of the strongest instruments for coffee intake, rs2472297, is also associated with smoking in the female population in UKBB (*P *=* *0.002; Supplementary Table S3, available as Supplementary data at *IJE* online). Therefore, when we identified an association between the GRSs of coffee intake that incorporated rs2472297 (i.e. the two sets of GRSs constructed using eight and six SNPs) and pregnancy outcomes, we conducted sensitivity analysis by including smoking as a covariate in the analysis.

A flowchart showing the number of individuals included in each analysis is presented in Supplementary Figure S2 (available as Supplementary data at *IJE* online).

##### ALSPAC

We constructed three weighted and three unweighted GRSs of coffee consumption as described above. We examined the association between the GRSs and maternal coffee intake (cups/day) at Week 32 during pregnancy in 6845 mothers. We also examined the association between the GRSs of coffee consumption and three pregnancy outcomes, with (*N* = 4510 for birthweight and 2989 for gestational age and pre-term birth) and without (*N* = 3714 for birthweight and 2456 for gestational age and pre-term birth) conditioning on child’s unweighted GRS or individual SNPs as described above in UKBB analysis. Covariates included maternal age at the last menstrual period and child’s sex. Analyses were conducted using a linear regression model for birthweight and gestational age, and a logistic regression model for pre-term birth.

Lastly, we examined the association between maternal GRS quartiles and two potential confounders (alcohol drinking and smoking during pregnancy) using chi-square tests.

A flowchart showing the number of individuals included in each analysis is presented in Supplementary Figure S3 (available as Supplementary data at *IJE* online).

### Associations between self-reported coffee consumption and pregnancy outcomes in the ALSPAC study

To explore whether previous observational associations regarding coffee consumption and birthweight were applicable to ALSPAC, we examined the phenotypic association between maternal coffee intake at Week 32 during pregnancy and the three pregnancy outcomes, including (i) child’s birthweight in 4302 mother–child pairs, (ii) weeks of gestation at birth, pre-determined by ALSPAC using estimated due date and (iii) pre-term birth defined as <37 weeks of gestation in 4231 mother–child pairs. We did not include analysis of stillbirth and miscarriage as we did not have information regarding coffee consumption in the pregnancy that ended in these outcomes.

Analyses were conducted using a linear regression model for birthweight and gestational age, and a logistic regression model for pre-term birth. Covariates included maternal age at the last menstrual period, maternal smoking and alcohol intake during pregnancy, and child’s sex.

A flowchart showing the number of individuals included in each analysis is presented in Supplementary Figure S3 (available as Supplementary data at *IJE* online). All statistical analyses were performed in R (version 3.4.3).

### Power calculations

We were interested in the statistical power of our study to detect the causal effect of maternal coffee consumption on different pregnancy outcomes. For the unconditional GRS analyses, we used the ‘Genetic Power Calculator’ (https://zzz.bwh.harvard.edu/gpc/qtlassoc.html).^34^ For risk of miscarriage, we used the MR power calculator (https://shiny.cnsgenomics.com/mRnd/).^35^ For all calculations, we assumed the absence of offspring genetic effects and a Type 1 error rate of α = 0.05 (the presence/absence of offspring genetic effects has little influence on the power to detect maternal genetic effects so long as the proportion of variance explained by the offspring genetic effects is small^36^). We estimated the size of the causal effect on each pregnancy-related outcome that we had 80% power to detect in our analyses. We note that whilst these asymptotic calculations assume slightly different underlying models/tests to those employed in our manuscript (e.g. the genetic power calculators test for association between the GRS and outcome assuming a certain size of causal effect of the exposure on the outcome; the MR power calculator uses a one-sample MR model to estimate asymptotic power, whereas we use two-sample MR in our study, etc.), the calculations should be good enough to provide useful approximations of the power of our study.

## Results

### Two-sample MR analyses

**Figure 2:**
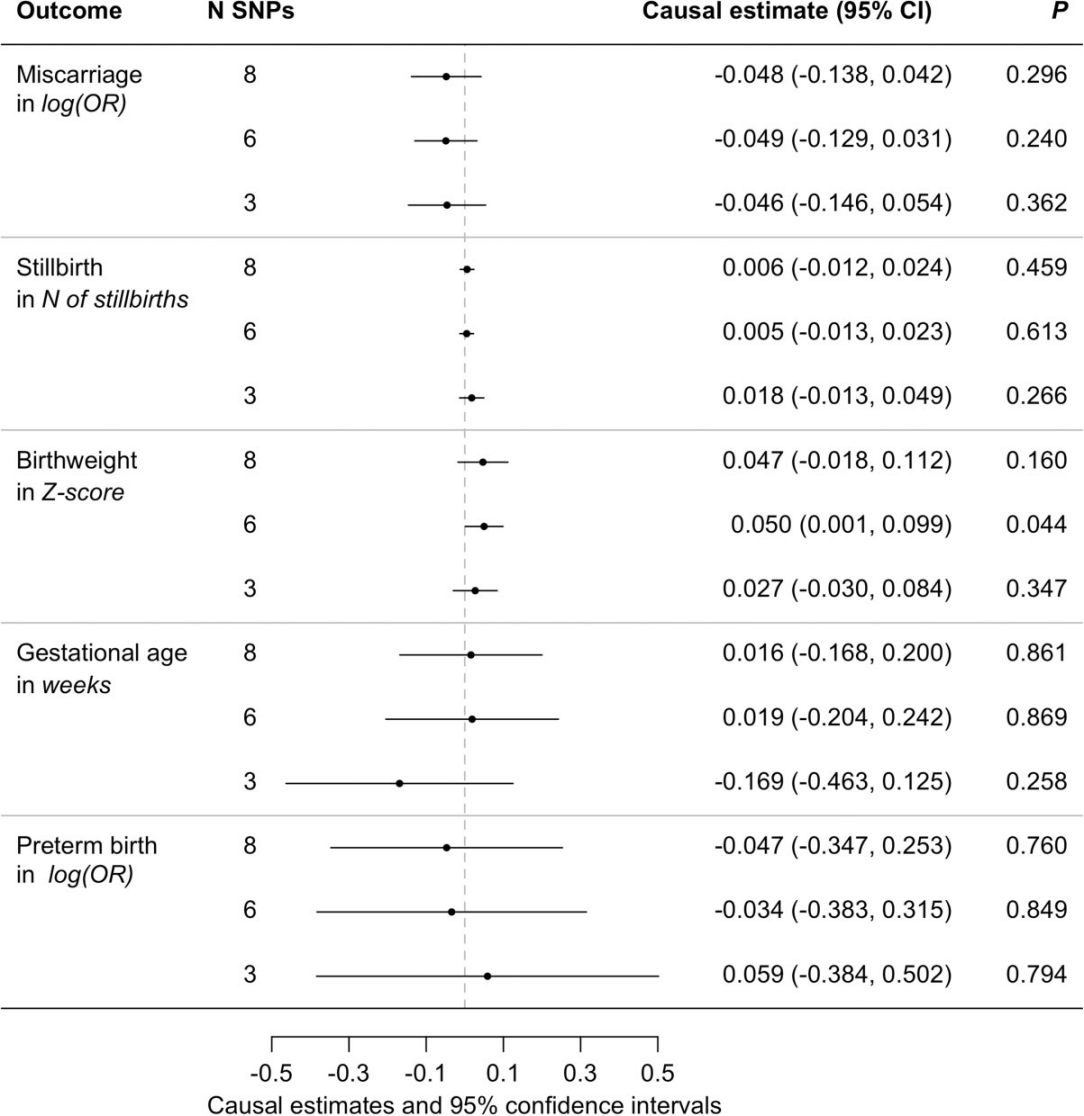
Overview of the effect estimates and 95% confidence intervals for the two-sample MR analyses per cup of coffee consumed per day. We did not observe a causal effect of coffee consumption (defined as cups per day) on any of the traits analyzed, although there was evidence of a small effect of increased coffee consumption on increased birthweight for the six SNP analysis. Birthweight was analyzed as a Z-score.^26^ Miscarriages were defined as the log(OR) of having had one to two spontaneous miscarriages [case control Genome Wide Association Study (GWAS)],^27^ stillbirth was defined as the number of self-reported incidents of stillbirth [http://www.nealelab.is/uk-biobank (10 December 2020, date last accessed)], gestational age was defined as self-reported length of gestation and preterm birth was defined as the log(OR) (log of the odds ratio) of having any birth at <37 weeks of gestation (case control GWAS).^28^ The units for the causal effects are per unit increase in the outcome per extra cup of coffee consumed per day. MR, Mendelian randomization; SNP, single nucleotide polymorphism.

We observed an effect of increased maternal coffee consumption on increased offspring birthweight in the analysis including six SNPs (IVW Effect size: 0.050 SD birthweight per cup of coffee/day, 95% CI: 0.001, 0.098) (Figure 2; Supplementary Table S5, available as Supplementary data at *IJE* online). The effects estimated using eight or three SNPs were in the same direction, but the effect sizes were attenuated. The 95% CIs around the estimated causal effect on the number of reported stillbirths, risk of sporadic miscarriages, pre-term births and gestational age overlapped zero. SNPs included in the GRSs are listed in Supplementary Table S2 (available as Supplementary data at *IJE* online).

Sensitivity analyses using MR–Egger, Weighted Median, Simple and Weighted Mode showed similar trends to the IVW estimates (Supplementary Table S5, available as Supplementary data at *IJE* online); however, due to the small number of variants, these sensitivity analyses are included only for completeness. The IVW heterogeneity analysis showed no strong evidence of heterogeneity in the causal effect estimate for coffee consumption on number of stillbirths, spontaneous miscarriage, gestational age, pre-term birth or birthweight, except for the birthweight analysis with all eight SNPs included in the model (heterogeneity analysis for IVW had a *P *=* *0.004). Likewise, we found no evidence of directional pleiotropy for maternal coffee consumption on any of the outcomes (Supplementary Table S6, available as Supplementary data at *IJE* online). However, due to the small number of variants in the analysis, the MR–Egger results should be carefully interpreted. The *I*^2^_GX_ of >0.99 was observed for all analyses, well above the cut-off of 0.9, which suggests little influence of weak instrument bias in the MR–Egger analysis.

### Individual-level data MR analysis using GRS (ALSPAC and UKBB)

#### UKBB

An overview of the demographics of the women included in the different analyses can be found in Supplementary Table S7 (available as Supplementary data at *IJE* online). The GRSs for coffee consumption were tested for association with the number of cups of coffee consumed per day in all female individuals from UKBB. An association was found between coffee consumption (*N* = 194 196) and the different unweighted and weighted GRSs (Supplementary Table S8, available as Supplementary data at *IJE* online), suggesting that having more coffee consumption increasing alleles identified in the sample of both men and women would increase a woman’s coffee consumption.

GRS analyses for the number of miscarriages (*N* = 78 681) and stillbirths (*N* = 78 867) showed no association (Table 1). An association between increased genetically predicted maternal coffee consumption and increased offspring birthweight was observed in the analyses using GRSs of eight and six variants (*N* = 155 242) (Table 1) and the association remained after adjusting for smoking in the sensitivity analysis.

**Table 1:** Results from the genetic risk score analysis for birthweight, miscarriage and stillbirth in UK Biobank

		Unweighted GRS			Weighted GRS		
		Effect estimate	SE	*P*	Effect estimate	SE	*P*
Miscarriage	8 SNPs	−0.003	0.002	0.225	−0.030	0.025	0.224
	6 SNPs	−0.004	0.003	0.079	−0.037	0.025	0.145
	3 SNPs	−0.001	0.004	0.699	−0.017	0.037	0.651
Stillbirth	8 SNPs	0.001	0.001	0.439	−0.001	0.008	0.894
	6 SNPs	0.000	0.001	0.807	−0.003	0.008	0.684
	3 SNPs	0.002	0.002	0.106	0.007	0.012	0.590
Birthweight	8 SNPs	2.64	0.698	**1 × 10^−4^**	30.5	7.98	**1 × 10^−4^**
	6 SNPs	2.21	0.805	**0.006**	27.4	8.25	**9 × 10^−4^**
	3 SNPs	1.66	1.15	0.148	20.09	12.00	0.094
Birthweight adjusted for smoking	8 SNPs	2.64	0.699	**1 × 10^−4^**	30.4	7.99	**1 × 10^−4^**
	6 SNPs	2.24	0.806	**0.006**	27.3	8.26	**9 × 10^−4^**

Results with *P *<* *0.05 are shown in bold. Birthweight is measured in grams. Miscarriages and stillbirth are the number of self-reported incidents. Unweighted GRS is coded as the number of alleles increasing coffee consumption, whereas in the weighted GRS the dosage of each SNP is weighted by the effect size reported in the original GWAS.^22^ All effect estimates denote the expected increase in the outcome per unit change in GRS. UKBB, UK Biobank; SNP, single-nucleotide polymorphism; SE, standard error; GRS, genetic risk score; GWAS, Genome Wide Association Study.

##### Mother–offspring pairs

To investigate whether adjusting for offspring GRS would impact our results, we performed the GRS analysis in a subset of individuals in UKBB where both maternal and offspring GRSs were available in addition to offspring self-reported birthweight (*N* = 2965). We were not able to detect any associations between maternal GRS and offspring birthweight in these analyses (Table 2).

**Table 2:** Mother–offspring pairs genetic risk score analysis for birthweight in UK Biobank

		Unweighted GRS			Weighted GRS		
		Effect estimate	SE	*P*	Effect estimate	SE	*P*
Maternal GRS	8 SNPs	2.44	4.24	0.565	50.4	49.0	0.303
	6 SNPs	4.81	4.94	0.330	61.9	50.9	0.224
	3 SNPs	−0.996	6.96	0.886	19.9	73.5	0.787
Maternal GRS, adjusting for offspring genetics	8 SNPs	5.32	4.96	0.284	94.1	57.4	0.101
	6 SNPs	8.34	5.74	0.146	106	59.3	0.072
	3 SNPs	1.80	7.99	0.822	53.4	84.8	0.529

Birthweight is measured in grams. For the weighted GRS analysis, individual SNPs in the offspring were added as covariates to the regression model, whereas an unweighted GRS for the offspring was used as a covariate in the unweighted analyses. All effect estimates denote the expected increase in birthweight per unit change in GRS. UKBB, UK Biobank; SNP, single-nucleotide polymorphism; SE, standard error; GRS, genetic risk score.

#### ALSPAC

An overview of the demographics of the women included in the different analyses can be found in Supplementary Table S7 (available as Supplementary data at *IJE* online). Both weighted and unweighted GRSs of maternal coffee consumption constructed using either eight or six SNPs were associated with increased coffee consumption at Week 32 of gestation (GRSs of eight and six SNPs account for 0.37% and 0.35% of the variance, respectively; *P ≤ *0.003) in ALSPAC. Although the GRSs constructed using three SNPs were not associated with coffee consumption during pregnancy (*P ****≥**** *0.457), the direction of the effect was the same but the effect size was attenuated. There was no association between any of the GRSs and maternal alcohol drinking or smoking during pregnancy (*P *>* *0.05; Supplementary Table S9, available as Supplementary data at *IJE* online).

Maternal GRSs were not associated with offspring birthweight, gestational age or pre-term birth either with or without adjusting for offspring genetics (Table 3). We also conducted the analysis without adjusting for offspring sex to mimic the conditions in UKBB and obtained similar results (Supplementary Table S10, available as Supplementary data at *IJE* online).

**Table 3:** Genetic risk score analysis of mother–offspring pairs for birthweight, gestational age and pre-term birth in ALSPAC

		Unweighted GRS			Weighted GRS		
		Effect estimate	SE	*P*	Effect estimate	SE	*P*
Birthweight, maternal GRS (*N* = 4510)	8 SNPs	0.134	3.505	0.970	−14.200	39.852	0.720
	6 SNPs	0.567	4.064	0.889	−13.238	41.128	0.748
	3 SNPs	0.121	5.844	0.984	−34.437	60.867	0.572
Birthweight, maternal GRS, adjusting for offspring genetics (*N* = 3714)	8 SNPs	−4.337	4.537	0.339	−55.786	51.429	0.278
	6 SNPs	−3.597	5.269	0.495	−51.005	53.559	0.341
	3 SNPs	−1.549	7.563	0.833	−59.261	78.710	0.452
Gestational age, maternal GRS (*N* = 2989)	8 SNPs	0.014	0.020	0.464	0.216	0.222	0.331
	6 SNPs	0.011	0.023	0.625	0.184	0.231	0.426
	3 SNPs	−0.005	0.033	0.885	−0.099	0.343	0.774
Gestational age, maternal GRS, adjusting for offspring genetics (*N* = 2456)	8 SNPs	0.007	0.025	0.769	0.012	0.282	0.966
	6 SNPs	0.005	0.029	0.873	0.007	0.295	0.981
	3 SNPs	−0.025	0.042	0.556	−0.427	0.434	0.326
Pre-term birth, maternal GRS (*N* = 2989)^a^	8 SNPs	−0.021	0.039	0.588	−0.537	0.445	0.228
	6 SNPs	−0.008	0.046	0.867	−0.4804	0.4645	0.301
	3 SNPs	0.031	0.066	0.641	0.021	0.686	0.976
Pre-term birth, maternal GRS, adjusting for offspring genetics (*N* = 2456)^a^	8 SNPs	0.004	0.052	0.945	0.124	0.598	0.836
	6 SNPs	−0.009	0.061	0.880	0.031	0.625	0.961
	3 SNPs	0.045	0.088	0.609	1.107	0.921	0.229

Birthweight reported in grams. Gestational age was reported in weeks. All effect estimates denote the expected increase in the outcome per unit change in GRS. Both maternal and offspring GRSs were coded by counting the number of coffee consumption increasing alleles. For the weighted GRS, each SNP was weighted by the effect size of the SNP. All analyses were adjusted for maternal age and offspring sex. ALSPAC, Avon Longitudinal Study of Parents and Children; *N*, number of individuals; SNP, single-nucleotide polymorphism; SE, standard error; GRS, genetic risk score.

a The effect estimates for pre-term birth are the log-transformed odds ratios.

### Associations between self-reported coffee consumption and pregnancy outcomes in the ALSPAC study

A higher maternal self-reported coffee consumption at Week 32 of gestation was associated with a lower offspring birthweight (effect size: −9.63 grams/cups, 95% CI: −17.57, −1.69); however, when adjusting for smoking and alcohol consumption during pregnancy, this association attenuated and the 95% CI overlapped zero (effect size: −4.52 grams/cups, 95% CI: −14.92, 5.88). Maternal self-reported coffee consumption was associated with a higher risk of pre-term birth both when adjusting for smoking and alcohol (odds ratio: 1.11, 95% CI: 1.03, 1.19) and without adjustment (odds ratio: 1.08, 95% CI: 1.02, 1.14). We found a similar trend for the association with gestational age with (effect size: −0.03 weeks/cups, 95% CI: −0.07, 0.01) or without adjusting for alcohol and smoking during pregnancy (effect size: −0.02 weeks/cups, 95% CI: −0.05, 0.01), even though their 95% CI overlapped zero.

### Power calculations

Supplementary Table S11 (available as Supplementary data at *IJE* online) shows approximate causal effect sizes that we have 80% power to detect in our main analyses. As expected, the power to rule out negative effects depends critically on the amount of variance explained in coffee consumption by our GRSs. In the case of conservative estimates for the amount of variance explained in coffee consumption (i.e. a GRS explaining 0.37% of the variance), we are well powered to rule out moderate to large adverse effects of coffee consumption on many pregnancy-related outcomes (i.e. coffee consumption explaining a 26.9-gram difference in offspring birthweight per cup of coffee per day, 0.03 more still births per cup of coffee per day, 0.09 more miscarriages per cup of coffee per day or 0.11 increased log odds of miscarriage per cup of coffee per day). Indeed, if the GRS explains a more substantial proportion of the variance in coffee consumption (i.e. ∼2%), then we are well powered to rule out the action of much smaller negative causal effects (Supplementary Table S11, available as Supplementary data at *IJE* online).

## Discussion

We did not find strong evidence for a causal effect of maternal coffee consumption on the number of miscarriages, stillbirths, gestational age or pre-term birth in any of our analyses conducted using either individual-level data from UKBB and ALSPAC or summary level data from 23andMe or publicly available sources. We constructed three sets of genetic instruments associated with coffee consumption in men and non-pregnant women, and found that the instruments containing either eight or six SNPs were associated with self-reported coffee consumption at Week 32 of pregnancy and therefore potentially good instruments for coffee consumption during pregnancy. Our results suggest previously reported observational associations^14–16^ may be driven by confounding factors such as smoking and alcohol consumption during pregnancy.^14^ Our results are consistent with a recent MR analysis by Yuan *et al.* using data from UKBB and the FinnGen Consortium, who also found no strong causal relationship between caffeine consumption and pregnancy loss.^37^ Yuan *et al.* define ‘pregnancy loss’ as a history of having stillbirth, spontaneous miscarriage or termination (UKBB), or as spontaneous abortion as classified by the International Classification of Diseases 8th to 10th codes (FinnGen). Our results confirm and extend the findings of Yuan *etal.*^37^ by investigating the effect of coffee consumption on risk of miscarriage and number of stillbirths, and by examining the relationship between coffee consumption and other perinatal outcomes (i.e. birthweight, gestational age, pre-term birth).

We were able to replicate the previously reported observational association between increased maternal coffee consumption during pregnancy and decreased offspring birthweight/higher risk of pre-term birth in ALSPAC, when we did not include smoking and alcohol as covariates. However, the observational association with birthweight attenuated when these variables were adjusted for, suggesting these factors may have confounded previous observational relationships. Additionally, our MR analyses did not support a causal relationship for either outcome.

We showed a causal effect of increased maternal coffee consumption on increased offspring birthweight in the analyses using either eight (both two-sample and one-sample analysis) or six SNPs (only one-sample analysis). The effect sizes were ∼0.05 for the two-sample MR, corresponding to an increase in birthweight of ∼24 grams per cup of coffee drunk each day, whereas the one-sample analysis showed effect sizes corresponding to an increase of ∼16 grams per coffee-drinking-increasing allele. In the analysis with eight SNPs, we included variants in both the *GCKR* gene and the *BDNF* gene known to be associated with type 2 diabetes and body mass index (BMI) and smoking behaviour, respectively. As expected, the SNP in the *GCKR* gene was associated with birthweight, whereas the SNP in the *BDNF* gene was associated with both smoking, BMI and birthweight. Both these variants were removed from the analysis when using either six or three SNPs. The inconsistency could also be partly due to the variance in coffee consumption explained by the three SNP GRSs being less than half that explained by the six or eight SNP GRSs. Additionally, these results could not be replicated in the mother–offspring analysis either in UKBB or in ALSPAC, although this could be due to the effect being too small to be detected with the substantially smaller sample size. Overall, the birthweight results should be interpreted with caution, as the results from the eight and six SNP GRSs could reflect pleiotropy from smoking or alcohol-related SNPs.

One of the strengths of our two-sample MR analysis is the use of partitioned maternal genetic effects from a birthweight GWAS, allowing estimation of the maternal genetic effects on birthweight conditional on the fetal genome and using these effects in the two-sample MR, as well as using conditional mother–offspring analysis for the GRS analysis of birthweight (UKBB and ALSPAC) and analysis of gestational age and pre-term birth (ALSPAC). However, these partitioned genetic effects are naturally not available for stillbirths and miscarriages, and we therefore could not perform MR analysis of the maternal contribution alone.

Our approach has a number of limitations, which we discuss in the remaining paragraphs. First, the SNPs used in the analysis were identified in the general population rather than in pregnant women. Nevertheless, we have shown that GRSs consisting of these SNPs are associated with coffee consumption in women during pregnancy, although the effect sizes on coffee consumption appear to be smaller in pregnant women in ALSPAC compared with non-pregnant women in UKBB. This could be a consequence of women drinking fewer cups of coffee during their pregnancy and genetic effect sizes being proportionally reduced.

Ideally, our genetic instruments would explain as much variance as possible in coffee consumption in order to have maximum power to estimate any causal effects of maternal coffee drinking on pregnancy-related outcomes. Variance in coffee consumption explained by the three SNPs was 0.82%, which is much lower than that explained by six SNPs [1.81% (or 0.35% in pregnant women in ALSPAC)] or eight SNPs [1.94% (or 0.37% in pregnant women in ALSPAC)]. The GRSs with three SNPs were associated with coffee consumption in non-pregnant women in the large UKBB but not with coffee consumption at gestational Week 32 in the smaller ALSPAC cohort, suggesting they are not good instruments for the ALSPAC analysis. However, analysis in ALSPAC showed that the GRS constructed of six SNPs had a strong association with coffee consumption in pregnancy (explaining 0.35% of the variance) and no association with drinking alcohol or smoking in pregnancy, suggesting that this set of SNPs might be the most appropriate to use in the context of coffee consumption in pregnancy.

Power analyses suggest that UKBB has 80% power (alpha = 0.05) to detect an effect of maternal coffee consumption equivalent to a 26.9-gram difference in birthweight per cup of coffee per day, or 0.03 more stillbirths per cup of coffee per day, if we conservatively assume that the coffee GRSs explain ∼0.37% of the variance in maternal coffee consumption during pregnancy. These asymptotic results and others suggest we have adequate power in our analyses to exclude large adverse effects of maternal coffee consumption on many pregnancy-related outcomes, but not the smaller magnitude causal effects that were estimated in this study. In this context it is also important to note we do not find strong evidence of an inverse effect of coffee consumption on birthweight, as previous observational analyses have suggested. Given our power calculations, we can be confident that a large inverse effect of coffee consumption on offspring birthweight is unlikely.

Lastly, although coffee is the primary source of caffeine in most populations^2^^,^^3^ other beverages such as tea and soft drinks or foods such as chocolate could contribute to an individual’s total caffeine intake. Importantly, GRS for coffee consumption have previously been shown to explain some of the variation in tea consumption in UKBB.^31^ This suggests that the effect observed could be partially mediated through tea consumption as well as coffee consumption, potentially through caffeine, as the GRSs were only associated with standard tea drinking, but not other forms of tea such as herbal or green tea.^31^ More detailed phenotyping in regard to actual caffeine consumption may be needed to properly proxy total caffeine intake.

Taken together we have performed a large MR study using three different sets of genetic variants in three cohorts, with additional publicly available data. All of our analyses show similar results and suggest that if any adverse effects of coffee consumption on birthweight, gestational age, pre-term birth, stillbirth or miscarriage exists, it is likely to be small.

## Conclusion

In conclusion, we did not find strong evidence of an adverse causal relationship between maternal coffee consumption and birthweight, gestational age, the number of stillbirths, risk of pre-term birth or spontaneous miscarriages. Notably, a null association may be due to a lack of power and needs to be validated in even larger studies.

## Ethics approval

This project received ethical approval from the Institutional Human Research Ethics committee, University of Queensland (Approval Number 2019002705).

## Supplementary Material

dyac121_Supplementary_Data

## Data Availability

UK Biobank (https://www.ukbiobank.ac.uk/) and ALSPAC (http://www.bristol.ac.uk/alspac/) data are available to researchers upon application to the individual cohorts via their websites.
