# Supplementary material for: Mendelian randomization study of maternal coffee consumption and its influence on birthweight, stillbirth, miscarriage, gestational age and pre-term birth
Source: Int J Epidemiol. 2022 Jun 9;52(1):165–77. doi: 10.1093/ije/dyac121 (PMC9908064; doi:10.1093/ije/dyac121)
Supplement: dyac121_Supplementary_Data [file dyac121_supplementary_data.zip › dyac121_Supplementary_Data/ije-2021-11-1719-File005.docx]

Supplementary data:

**Figure S1: Flowchart showing the sample size for the two-sample MR**

**analysis.**

**Figure S2: Flowchart showing the sample size for each analysis in UKBB.**

**
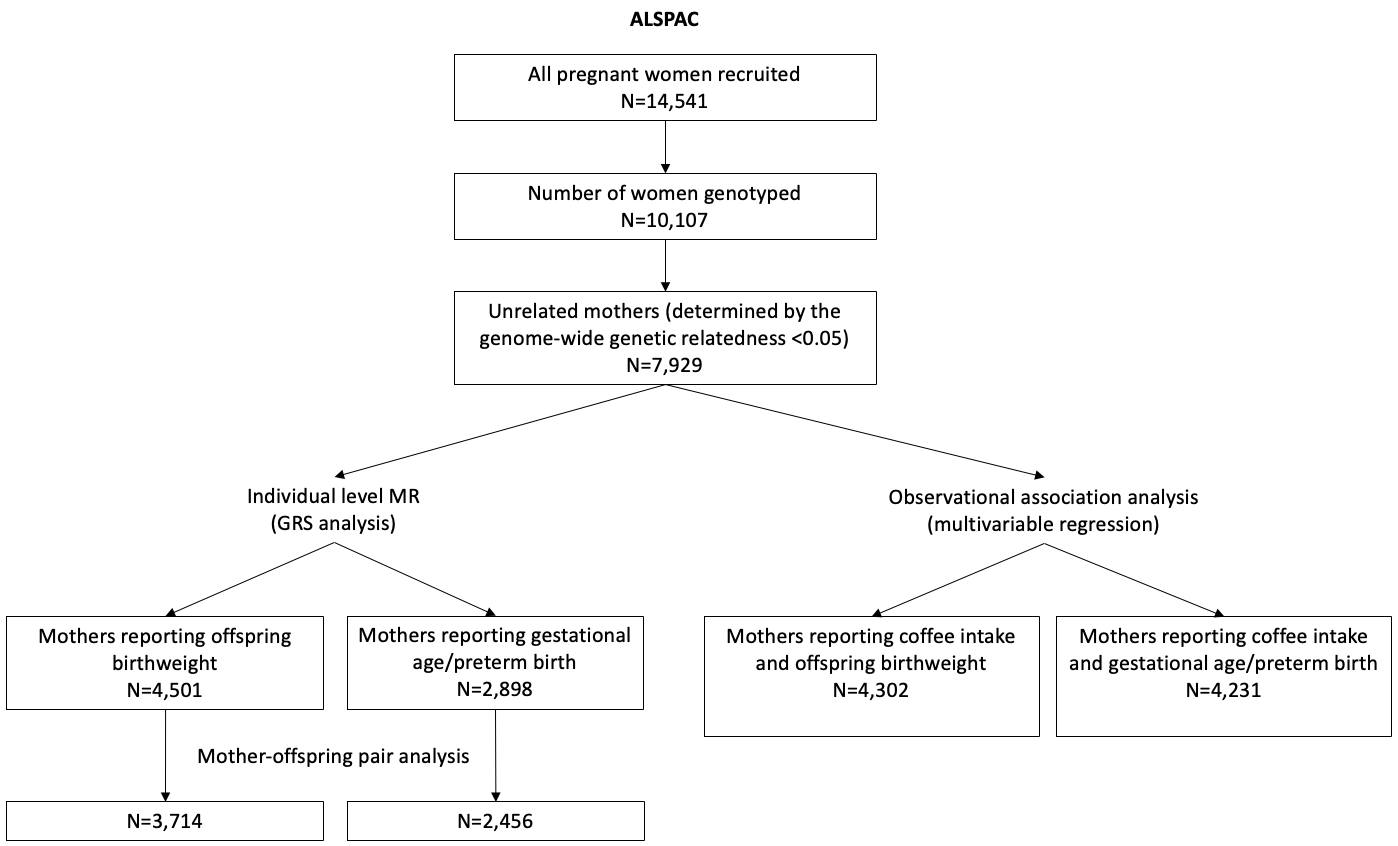
**

**Figure S3: A flowchart showing the sample size for each analysis in the ALSPAC.**

**Note S1: UKBB European individuals**

We used imputed genetic data from the October 2019 release from the UKBB(1) for our analyses (Application ID: 53641). In addition to quality control metrics performed centrally by the UKBB(1), we defined a subset of unrelated individuals of European ancestry. This has been described in detail elsewhere(2). Briefly, we generated ancestry informative principal components from the 1000 genomes samples(3). The UKBB participants’ ancestry was projected onto these components and classified using K-means clustering centered on the three main 1000 genomes populations (European, African, and South Asian). Those clustering with the European cluster were classified as having European ancestry. Only participants reporting as “British”, “Irish”, “White” or “Any other white background” were included in the clustering analysis. Second, to identify a subset of unrelated individuals, we generated a genetic relationship matrix in the GCTA software package(4) (version 1.90.2) and excluded one of every pair of related individuals with a genome-wide genetic relationship greater than 9.375%. Individuals who had withdrawn consent from UKBB as of February 2021 were also excluded from analyses.

**Note S2: UKBB phenotypes**

Coffee consumption for the UKBB women (N=193,948) was defined as the number of cups of coffee drunk per day (Data field: 1498; Q: How many cups of coffee do you drink each day?), and data from individuals who did not report a number (i.e., other options were ‘prefer not to say’, ‘do not know’) or if they did not answer the questions were excluded from our analysis. Women who reported ‘less than one cup per day’ were coded as 0.

Individuals who reported their smoking behavior (N=193,508) (Data field: 20116) were coded in the UKBB as 0 if they never smoked, 1 if they previously smoked and 2 if they were current smokers. Those who reported ‘prefer not to say’, ‘do not know’ or did not provide any information were excluded from our analysis. Alcohol intake frequency (Data field: 1558, Q: About how often do you drink alcohol?) was also obtained in UKBB (N=194,097). People who reported that they drink alcohol daily were coded in the UKBB as ‘5’, those who drink 3 to 4 times a week were coded as ‘4’ and those who drink 1 to 2 times a week as ‘3’. If the individuals reported drinking only 1 to 3 times a month then they were assigned ‘2’ and ‘1’ if they reported drinking only on special occasions. The variable was coded as ‘0’ if the women reported they never drank alcohol. Data were excluded from the analysis if the individual reported ‘prefer not to say’, ‘don’t know’ or if they did not answer the question.

A subset of women in the UKBB (N= 87,825) have answered the “Female-specific factors” category where how many spontaneous miscarriages and stillbirths they had were recorded. Responses were excluded from the analysis for those who reported ‘prefer not to say’, ‘don’t know’ or if they did not provide any information for the stillbirth (N=115,329) and spontaneous miscarriage data collection (N=115,515), yielding an N =78,867 for the stillbirth analysis and N = 78,681 for the miscarriage analysis. The birthweight of their first child (in pounds) was also reported (N=173,259) along with their age at first live birth (N=155,242). Women who reported ‘prefer not to say’, ‘do not know’, ‘only had twins’ or if they did not answer the question for the first offspring birthweight data (N=20,937) or for the age at first live birth (N=38,954) were not included in the analysis.

**Note S3: ALSPAC cohort**

The study recruited 14,541 pregnant women in a defined area in the South West of England with an expected delivery date between April 1991 and December 1992. Of these initial pregnancies, there was a total of 14,676 foetuses, resulting in 14,062 live births and 13,988 children who were alive at 1 year of age. After the oldest children were approximately seven years of age, there were 913 new pregnancies recruited during Phases II, III and IV of enrolment, resulting in an additional 913 children being enrolled. The total sample size for analyses using any data collected after the age of seven is therefore 15,454 pregnancies, resulting in 15,589 foetuses. Of these 14,901 were alive at 1 year of age. The mothers and their children have been followed up through postal questionnaires and at clinics. Study data were collected and managed using REDCap electronic data capture tools hosted at the University of Bristol. REDCap (Research Electronic Data Capture)(5) is a secure, web-based software platform designed to support data capture for research studies. Please note that the study website contains details of all the data that is available through a fully searchable data dictionary and variable search tool and reference the following webpage: <http://www.bristol.ac.uk/alspac/researchers/our-data/>. Mothers and children were genotyped using the Illumina Human660W-quad array and the Illumina HumanHap550 quad chip genotyping platform, respectively. Imputation was performed using Impute V2.2.2 against the 1000 genomes phase 1 version 3 reference panel. In total, 10,107 mothers were genotyped(6). However, only unrelated mothers (N=7,929), determined by a genome-wide genetic relatedness smaller than 0.05 (one individual per related pair was removed), were included in the following analyses. Ethical approval for the study was obtained from the ALSPAC Ethics and Law Committee and the Local Research Ethics Committees.

**Note S4: UKBB mother-offspring pairs**

It is important to consider that the maternal genome is correlated 0.5 with the offspring genome(7, 8). This means that using maternal SNPs in MR studies of maternal exposures and offspring outcomes without conditioning on offspring genotype could violate core assumptions relating to horizontal pleiotropy (9) and complicate interpretation of results. We, therefore, leveraged the mother-offspring pairs in the UKBB. Pairwise kinship estimation was performed centrally on the whole UKBB cohort using the KING software(10). Parent-offspring pairs were identified using the software defaults for the estimated kinship coefficients (φ) and IBS0 cut-offs(1, 10). For a given parent offspring pair, the individual that was the parent (in this case the mother) and the individual that was the offspring was determined using their reported sex and date of birth. Offspring who self-reported being part of a multiple birth were excluded from analyses. Only the eldest offspring from a singleton birth who had available data was included in the analysis when mothers who had multiple offspring in the UKBB were detected. We only retained one of any related pair of mothers (who was less that 3rd-degree relatives defined by KING software, i.e. φ < 6.25%) in the remaining pairs (10). There were 3,953 mother-offspring pairs of European ancestry available, of which 3,134 pairs had offspring self-reported birthweight available. After removing children with birthweight smaller than 2.5 kg or greater than 4.5 kg to match the inclusion criterion used in the GWAS of birthweight, a total of 2,965 pairs were available for further analysis.

**References:**

1. Bycroft C, Freeman C, Petkova D, et al. The UK Biobank resource with deep phenotyping and genomic data. Nature. 2018;562(7726):203-9.

2. Warrington NM, Hwang L-D, Nivard MG, Evans DM. Estimating direct and indirect genetic effects on offspring phenotypes using genome-wide summary results data. Nature Commun. 2021;12(1):5420.

3. Auton A, Abecasis GR, Altshuler DM, et al. A global reference for human genetic variation. Nature. 2015;526(7571):68-74.

4. Yang J, Lee SH, Goddard ME, Visscher PM. GCTA: a tool for genome-wide complex trait analysis. Am J Hum Genet. 2011;88(1):76-82.

5. Harris PA, Taylor R, Thielke R, Payne J, Gonzalez N, Conde JG. Research electronic data capture (REDCap)--a metadata-driven methodology and workflow process for providing translational research informatics support. J Biomed Inform. 2009;42(2):377-81.

6. Fraser A, Macdonald-Wallis C, Tilling K, et al. Cohort Profile: the Avon Longitudinal Study of Parents and Children: ALSPAC mothers cohort. Int J Epidemiol. 2013;42(1):97-110.

7. Brumpton B, Sanderson E, Hartwig FP, et al. Within-family studies for Mendelian randomization: avoiding dynastic, assortative mating, and population stratification biases. bioRxiv. 2019:602516.

8. Evans DM, Moen GH, Hwang LD, Lawlor DA, Warrington NM. Elucidating the role of maternal environmental exposures on offspring health and disease using two-sample Mendelian randomization. Int J Epidemiol. 2019;48(3):861-75.

9. Didelez V, Sheehan N. Mendelian randomization as an instrumental variable approach to causal inference. Stat Methods Med Res. 2007;16(4):309-30.

10. Manichaikul A, Mychaleckyj JC, Rich SS, Daly K, Sale M, Chen W-M. Robust relationship inference in genome-wide association studies. Bioinformatics. 2010;26(22):2867-73.
